# Sutures versus staples for wound closure in orthopaedic surgery: a pilot randomized controlled trial

**DOI:** 10.1186/1754-9493-7-6

**Published:** 2013-02-09

**Authors:** Jesse A Slade Shantz, James Vernon, Saam Morshed, Jeff Leiter, Gregory Stranges

**Affiliations:** 1 Orthopedic Trauma Institute, University of California, 2550 23rd Street, Building 9, 2nd Floor, 94110, San Francisco, CA, USA; 2 Section of Orthopedics, University of Manitoba, AD-401, 820 Sherbrook St., R3A 1R9, Winnipeg, MB, Canada; 3 Pan Am Clinic, 75 Poseidon Bay, R3M 3E4, Winnipeg, Manitoba, Canada

**Keywords:** Sutures, Staples, Wound closure, Wound complication, Time of closure, Pain with removal

## Abstract

**Background:**

In the spectrum of surgical decision-making, wound closure material is often an afterthought. However, the findings of a recent meta-analysis suggest that the rate of surgical site infections (SSIs) is increased by using staples to close surgical wounds. Less clear is the effect of closure material on the incidence of non-infectious wound complications.

The aim of this study was to compare sutures and staples in terms of: incidence of wound complications to determine the sample size for a definitive trial comparing wound closure methods.

**Methods:**

Eligible adult orthopaedic patients were randomized to have wounds closed with sutures or staples. Time for skin closure was recorded. Wounds were assessed for complications for six weeks. The incidence of complications was compared using Fisher’s exact test. Time to close and pain with removal of closure material were compared using a Student’s t-test.

**Results:**

The total number of patients reporting a wound complication was 59 of 148 patients completing six-week followup (41%), with no differennce between sutures and staples (RR = 0.77, CI = 0.52–1.14). The time to close wounds was shorter in the staple group (mean=4.8 min, CI = 2.6–7.1) than the suture group (mean=12 min, CI = 7.9–16). Patients in the staple group (mean=3.7, CI =2.8–4.6) reported more pain with removal than suture group (mean=2.5, CI =1.6–3.4).

**Conclusions:**

This study suggests that 42% of patients report a wound complication with no difference between sutures and staples. It was demonstrated that suturing skin requires more time and staples are more painful to remove.

**Trial registration:**

Clinicaltrials.gov identifier NCT01146236 (registered June 14, 2010)

## Background

Surgical site infections (SSIs) are one of the most common, and most important, nosocomial infections in post-operative patients. Two-hundred and ninety-thousand SSIs occur in the United States annually following elective orthopedic surgery resulting in $1 billion to $10 billionadditional healthcare costs according to CDC estimates [1]. Orthopedic SSIs can prolong hospital stay by a median of two weeks per patient and increased healthcare costs by more than 300% per case [2]. A potentially more insidious post-operative occurrence are the minor complication(s) faced by patients related to their wound closure. Few research studies address the burden that these complications such as wound drainage, wound necrosis, dehiscence and blistering place on patients, office staff and the healthcare system as a whole.

Most surgeons choose a method of closure based on training, past experiences, and convenience. A recent meta-analysis comparing staples to sutures in orthopaedic wound closure demonstrated a three-fold increase in infections in stapled wounds compared with sutured wounds [3]. The ensuing debate surrounding the results of this meta-analysis has pointed out that the included studies [4-9] did not uniformly employ experimental designs that limit bias. Additionally, the included studies employed heterogeneous outcome definitions drawing into question the generalizability of the meta-analysis.

Animal studies have shown the mechanical equivalence of stapled and sutured wounds [10], and clinical studies in several specialties have also failed to show superiority of the cosmetic appearance of stapled or sutured wounds [11,12]. However, no comparative trial exists that has the statistical power to determine if the rate of infection in wounds closed with staples is different from that in wounds closed with sutures in orthopedic procedures. More importantly, great uncertainty exists surrounding the incidence of all infectious and non-infectious wound complications after open orthopaedic procedures. Available studies report rates of between 13% and 60% [8,13-15].

The aim of this pilot study was to: determine the sample size required to definitively prove the superiority of sutures or staples for wound closure in orthopaedic surgical procedures in terms of reducing wound complications.

## Methods

**Figure 1:**
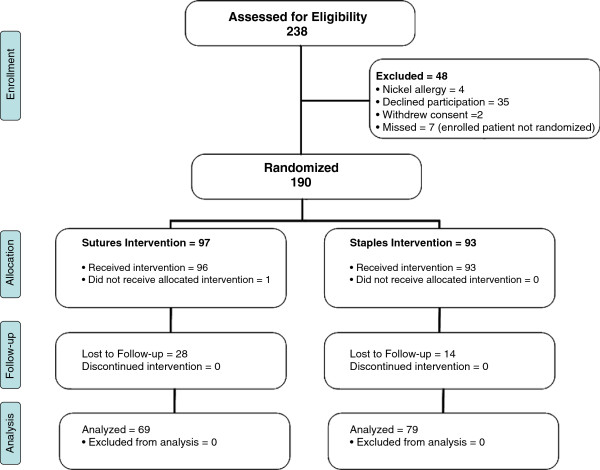
Schematic of Study Design.

All adult orthopaedic patients undergoing a non-emergent procedure between December 2010 and November 2011 were screened for eligibility either in the preadmission clinics for elective cases or after admission for trauma-related procedures. Patients were consented for participation prior to screening. Patients were included if they were to undergo an orthopedic procedure requiring a wound greater than two centimeters in length. Exclusion criteria included, open fractures, procedures of the foot or hand, arthroscopic procedures and chemotherapy or radiation therapy treatment. The study was approved by the local Human Research Ethics Board. A schematic of the trial design can be found in Figure 1.

Baseline parameters including age, weight, height were collected. Medical comorbidities affecting wound healing including: diabetes, peripheral vascular disease, renal failure requiring dialysis, liver failure and inflammatory arthritis were identified. Medications including corticosteroids, transplant anti-rejection medications, disease-modifying anti-rheumatic drugs warfarin, asprin, clopidogrel and heparin were noted by patient report. A smoking history was also obtained. A trained research assistant or orthopaedic surgery resident collected patient data on case report forms which were later entered into an electronic database (MS Excel, Microsoft, Redmond, WA).

Allocation of patients to treatment groups occurred at the completion of deep wound closure using sequentially-numbered opaque envelopes with randomization sequence generated by the principle investigator using Microsoft Excel (Redmond, WA). Any participant felt to be endangered by the use of one closure method was withdrawn from the study prior to allocation.

After completion of the procedure, deep tissues were closed with absorbable braided suture (Polysorb, Covidien, Mansfield, MA). In all patients the subcutaneous tissue was also closed with an absorbable braided suture. Patients allocated to the sutures intervention had their wounds closed using the suture material chosen by the primary surgeon. The primary surgeon also decided on the most appropriate technique of closure. Those allocated to the staples group were closed using a commercially-available stapler (Weck Visistat 35W, Limerick, PA). Closure material was removed, when necessary, during a wound check two weeks after surgery by medical staff or orthopedic technologists.

Patients were blinded to treatment allocation by use of an adhesive bandage or plaster which remained in place until the first planned post-operative visit when the closure material was removed or the wound was checked, including the removal of steristrips in the case of absorbable subcuticular closure. During closure material removal the wound was hidden from the view of the patient when necessary to maintain blinding. Immediately prior to unblinding patients completed an outcome assessment survey including a questionnaire determining complication occurance and a 100 mm VAS pain scale. Pain measurements were normalized to worst pain imaginable reported by the patient.

Wound complications were identified by hospital staff during the post-operative inpatient stay and were reported by patients at two- and six-week follow-up appointments in a survey conducted by the study coordinator. Patients were asked whether they had taken antibiotics, required dressing changes or a re-operation at two- and six-week follow-up appointments, with affirmative answers resulting in further questioning to confirm the type of complication that had occurred. Suspected infections were defined as: reoperation, use of intravenous antibiotics or patient report of use of oral antibiotics related directly to the surgical procedure. Wound drainage, necrosis, dehiscence and suture abscess were suspected when dressing changes or additional medical treatment was reported by the patient. The hospital and clinic charts of all suspected infections and five negative controls were reviewed by an orthopaedic surgeon to confirm the diagnosis of surgical site infection as defined by the CDC. Secondary outcome measures of time taken to close wounds and pain with wound closure removal were also collected.

The primary outcome was a composite measure of all-cause, patient-reported wound complications. Primary outcome data for the entire cohort of patients encompassing four anatomic sites was analyzed using the Fisher’s exact test (Stata, College Station, TX) and are presented as relative risk and 95% confidence intervals. Strata-specific relative risks were calculated for each anatomic site using the primary outcome measure. Continuous secondary outcome measures were analyzed using a 2-tailed Student’s t-test. The results were reported as per the guidelines of the CONSORT group [16].

## Results

**Figure 2:**
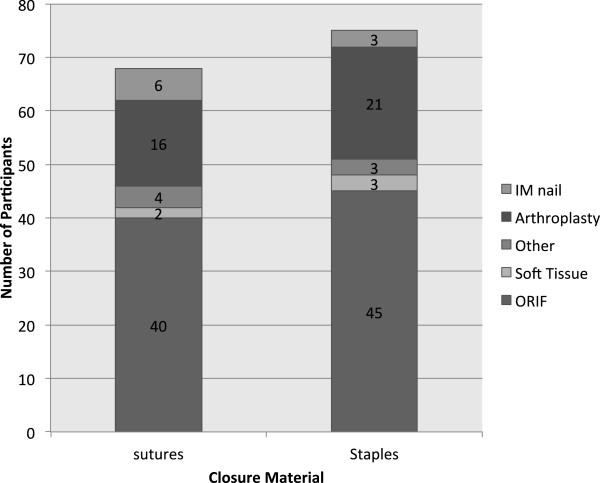
Types of Procedures Performed on Participants.

A total of 148 patients were randomized, 69 in the staples group and 79 in the sutures group. There was no significant difference in baseline parameters between the two randomized groups. The characteristics of patients and anatomic locations of the surgical sites are summarized in Table 1. The types of procedures performed are presented in Figure 2. In the sutures group wounds were closed with: absorbable running subcuticular (66%), interrupted non-absorbable horizontal matress (22%), interrupted non-absorbable vertical matress (6.2%) and other suturing techniques (6.2%).

**Table 1:** Characteristics of study participants

***Characteristic***	***Sutures (n=69)***	***Staples (n=79)***
Age (yrs)	52.3 (19–93)	52.3 (17–92)
Body Mass Index (kg/m^2^)	28.0 (14–47)	27.8 (17–43)
Medical Comorbidity	12(17%)	14(18%)
Smoking	21(30%)	21(27%)
Immunosuppressive	2(3%)	8(10%)
Anticoagulant	13(19%)	14(18%)
Anatomic Locations		
Upper Extremity	20(29%)	25(32%)
Hip	7(10%)	15(19%)
Knee	18(26%)	23(29%)
Ankle	24(35%)	16(20%)

There was no difference between the groups in mean surgical time (81.4; 95% C.I. 69.6–93.2 versus 79.7; 95% C.I. 69.0–90.5 min) (Table 2). However, the time to close wounds was significantly shorter in the staples group compared to the sutures group (4.8; 95% C.I. 2.6–7.1 versus 12; 95% C.I. 7.9–16 min). Expressed as a percentage of the total operative time the difference in closure time remains significant between the sutures and staples groups (7.9%; 95% C.I. 4.7–11 versus 15%; 95% C.I. 11–19).

**Table 2:** Comparison of skin closure methods by operative time and incidence of wound complications and infections

***Variable***	***Sutures***	***Staples***
Surgical time (min)	81.4 (69.6–93.2)	79.7 (69.0–90.5)
Closure time (min)	4.8 (2.6–7.1)	12 (7.9–16)
% total procedure	7.9% (4.7–11)	15% [10-18]
Wound Complication		
Absolute :	34	25
Percentage:	49%	32%
Suspected Infections		
Absolute :	19	14
Percentage:	28%	18%
Confirmed Infections	1	1
Pain with removal (2 wk)	2.5 (1.6–3.4)	3.7 (2.8–4.6)
Pain with removal (6 wk)	3.4 (2.5–4.3)	3.0 (2.1–3.9)
Data presented as Mean (95% CI) or Absolute Number		

The total number of patients reporting a wound complication was 59 (41%) (Table 2). There was no difference in the incidence of wound complications between the sutures (34 participants) and staples (25 participants) group (RR = 0.77, CI = 0.52–1.14). Stratification of the results by surgical site or type of procedure revealed no difference between groups (Table 3).

**Table 3:** Relative risk of wound complications for subgroups by anatomic location or procedure type

	***Relative risk***	***95% CI***
Overall	0.77	0.52–1.14
Anatomic Site		
Upper Extremity	1.04	0.53–2.03
Hip	0.86	0.41–1.79
Knee	0.07	0.31–1.57
Ankle	0.75	0.28–2.03
Procedure Type		
ORIF	0.70	0.40–1.22
Arthroplasty	0.81	0.37–1.80
IM nail	0.50	0.12–2.00
Soft tissue repair/reconstruction	0.75	0.15–3.72
Other	2.25	0.41–12.28

There were 33 (22%) suspected infections within the six-week follow-up period. Review of the charts of all suspected infections confirmed two infections, one in a sutured wound and one in a stapled wound. There was no difference in the rate of suspected SSI between the sutures (18) and staples (13) group (RR=0.77, CI=0.42–1.41). At 2-week follow-up, patients in the staples group reported more pain with removal than the sutures group 3.7 (95% C.I. 2.8–4.6) versus 2.5 (95% C.I. 1.6–3.4). However, by six weeks follow-up the difference in recalled pain was no longer significant.

## Discussion

The incidence of wound complications after orthopaedic surgery is not well defined. SSIs cause disability and increase treatment costs exponentially [2]. However, the impact of non-infectious complications has not yet been well defined. The preceding pilot study aimed to determine the number of patients necessary to determine the safest wound closure material for a variety of orthopaedic procedures. The time to close wounds and pain with removal of closure material were also compared.

This study found a patient-reported wound complication rate of 41% (59/148). Previous studies comparing sutures and staples have demonstrated rates of infection ranging from 0.04% to 13% [4-9]. The rate of confirmed SSIs in this study was 1.4% (2/148). It must be noted that 22% (33/148) of patients reported the use of antibiotics or further surgery related to their index procedure. Based on the results of this pilot study it is estimated that a sample size of 1100 patients would be necessary to show a 25% risk reduction in wound complications with 80% power for the combined group of surgical sites.

Studies have historically relied on a clinician’s interpretation of a wound complication, and have not included minor events, despite the importance of these events to the patient [4,6,9,18]. Therefore, a direct comparison of our results is difficult due to the lack of a definition of a wound complication. Although this rate was somewhat higher than expected, it has been shown that the rate of non-infectious wound complications in clean orthopedic and trauma surgery can be as high as 60% (630/1,073) [15]. Another prospective hip and knee arthroplasty patients found a wound complication rate of 32% (32/165) including a 9.7% (16/165) suspected infection rate [8]. Patel *et al.* also showed that over 50% (n=1437) of arthroplasty patients had persistent wound drainage on post-operative day four and further found that drainage increased hospital stay and was positively correlated with early SSI [14].

Many surgeons cite a speed-of-closure argument when justifying their use of staples over sutures despite the myriad of papers questioning this belief [3,4,6,8,9,19]. The mean difference for time of closure in this study indicates a seven minute time savings with the use of staples to close skin. If the average orthopaedic surgeon performs five procedures a day, the use of staples could save as much as 35 minutes of operative time preventing the cancellation of surgeries.

This study showed a small, clinically relevant difference in pain upon staple removal compared to that of suture removal.

There are several limitations to the results presented here. This pilot study represents a short-term follow-up of surgical wounds with only six-week data collected. Therefore, the actual incidence of wound complications may be underestimated. Previous studies comparing the incidence of wound complications in sutured and stapled wounds have used a restricted risk period between ten days and one year with 14 days being the most common duration. Future studies need to standardize the period for which attributable risk is considered. The loss to follow-up we experienced was also a significant concern, with over 20% of patients not completing the six-week study.

## Conclusions

In conclusion, this study suggests that there is a discrepancy between surgeon and patient expectations for the course of normal wound healing. Although much of the literature reports complication rates greater than 10% this prospective study suggests that 42% of open orthopaedic surgeries are associated with a patient-reported wound complication. This study suggests approximately 1100 participants are required to show a 25% risk reduction between sutures and staples in terms of wound complications.

## Abbreviations

SSI: Surgical site infections;CDC: Centers for disease control

## Competing interests

The authors do not have any competing interests related to the performance of this study.

## Authors’ contributions

All listed authors participated in the design of the above trial. JV, JSS and SM completed the data analysis. JV and JSS prepared the manuscript and literature review. SM, GS and JL reviewed this manuscript in its entirety. All authors read and approved the final manuscript.
